# The Emerging Clinical Significance of the Red Cell Distribution Width as a Biomarker in Chronic Obstructive Pulmonary Disease: A Systematic Review

**DOI:** 10.3390/jcm11195642

**Published:** 2022-09-25

**Authors:** Angelo Zinellu, Arduino A. Mangoni

**Affiliations:** 1 Department of Biomedical Sciences, University of Sassari, 07100 Sassari, Italy; 2 Discipline of Clinical Pharmacology, College of Medicine and Public Health, Flinders University, Sturt Road, Bedford Park, SA 5042, Australia; 3 Department of Clinical Pharmacology, Flinders Medical Centre, Southern Adelaide Local Health Network, Flinders Drive, Bedford Park, SA 5042, Australia

**Keywords:** red blood cell distribution width, biomarker, chronic obstructive pulmonary disease, acute exacerbations, diagnosis, prognosis

## Abstract

There is an intense focus on the identification of novel biomarkers of chronic obstructive pulmonary disease (COPD) to enhance clinical decisions in patients with stable disease and acute exacerbations (AECOPD). Though several local (airway) and circulatory inflammatory biomarkers have been proposed, emerging evidence also suggests a potential role for routine haematological parameters, e.g., the red cell distribution width (RDW). We conducted a systematic literature search in PubMed, Web of Science, and Scopus, from inception to April 2022, for articles investigating the diagnostic and prognostic role of the RDW in stable COPD and AECOPD. The risk of bias was assessed using the Joanna Briggs Institute Critical Appraisal Checklist. Significant associations between the RDW and the presence and severity of disease, outcomes (mortality, hospital readmission), and other relevant clinical parameters (right heart failure, pulmonary arterial hypertension) were reported in 13 out of 16 studies in stable COPD (low risk of bias in 11 studies), and 17 out of 21 studies of AECOPD (low risk of bias in 11 studies). Pending further research, our systematic review suggests that the RDW might be useful, singly or in combination with other parameters, for the diagnosis and risk stratification of patients with stable COPD and AECOPD (PROSPERO registration number: CRD42022348304).

## 1. Introduction

Despite the ever-increasing global public health and financial burden of chronic obstructive pulmonary disease (COPD), considerable challenges remain in the capacity of healthcare professionals to accurately diagnose the disease, particularly during the early stages, and to predict trajectories of lung function decline, acute exacerbations (AECOPD), and other adverse clinical outcomes [1]. Contrary to other pathologies, e.g., cancer, these uncertainties have curtailed the development and implementation of a personalised medicine approach to optimise disease control and improve quality of life in patients with COPD [2]. However, they have stimulated the search for novel biomarkers that may complement the clinical information currently gathered from physical examination, lung function tests, and imaging procedures [3]. The established role of infection, inflammation, and immunity in the pathophysiology and the clinical progression of COPD has led to the identification of a number of biomarkers within these pathways, e.g., eosinophils, procalcitonin, neutrophil elastase, and serum amyloid A, which can be characterised from different biological matrices, blood, saliva, or sputum [3,4,5,6]. Though some of these biomarkers, e.g., eosinophils and procalcitonin, appear particularly promising to enhance clinical and therapeutic decisions in patients with stable COPD and AECOPD [7], the availability of additional biomarkers from routine laboratory tests might also further improve patient care in those settings that have limited access to expensive and complex analytical facilities. In this context, studies have reported the diagnostic and prognostic potential of other blood cell types and derived parameters from routine laboratory tests, e.g., the neutrophil-to-lymphocyte ratio and the platelet-to-lymphocyte ratio, in patients with stable COPD and AECOPD [8,9,10,11]. A further routine haematological parameter currently under investigation as a potential biomarker is the red cell distribution width (RDW), an index used in the assessment of red blood cell morphology and anaemias that is calculated by dividing the standard deviation of red blood cell volumes by the mean corpuscular volume of the erythrocytes, and then multiplied for 100 [12,13]. Less commonly, another measure, the standard deviation of the RDW (RDW-SD), is used to express the arithmetic width of the red blood cell distribution curve measured at the 20% frequency level [14]. Elevations in RDW values, expressed as a percentage, indicate an increased heterogeneity in the volume of circulating red blood cell, a condition known as anisocytosis. The latter can be caused either by a reduced clearance of older red blood cells and/or by an impaired red blood cell production in the bone marrow [12,13]. Though an increase in the RDW has been reported in several physiological and pathological conditions [12,15,16,17,18,19,20,21,22,23], studies conducted over the last decade have also investigated the diagnostic and the prognostic role of the RDW specifically in patients with COPD. The aims of this systematic review were: (a) to critically appraise the available evidence regarding possible differences in RDW values between patients with stable COPD and AECOPD and control groups; and (b) to investigate the associations between the RDW and clinical outcomes and other relevant clinical parameters in these groups.

## 2. Materials and Methods

We conducted a systematic literature search for articles published in PubMed, Web of Science, and Scopus, between inception and the 15 April 2022, using the following terms (and their combination): “RDW” or “red blood cell distribution width” and “COPD” or “chronic obstructive pulmonary disease” or “AECOPD” or “acute exacerbation chronic obstructive pulmonary disease”. Abstracts and, if relevant, full articles were independently reviewed by two investigators. Handsearching citation lists of relevant articles was also performed to identify additional studies. The inclusion criteria for study selection were: (a) full-text available; (b) English language used; (c) description of associations between the RDW and either diagnosis, clinical outcomes, or other relevant clinical parameters in patients with COPD and/or AECOPD. The following parameters were captured from each study: age, sex, year of publication, country where the study was conducted, study design (prospective or retrospective), criteria use for the diagnosis of COPD, main comorbidities, established parameters for the assessment of the RDW in the general population (i.e., mean corpuscular volume, haemoglobin, vitamin B_12_, and folic acid), main results, and other significant associations between the RDW and clinical parameters. The risk of bias was assessed using the Joanna Briggs Institute (JBI) Critical Appraisal Checklist for case-control studies. Studies satisfying ≥75% of checklist items were considered as having a low risk [24]. The study followed the PRISMA 2020 statement on the reporting of systematic reviews (see Supplementary Tables S1 and S2) [25]. The protocol was registered in the International Prospective Register of Systematic Reviews (PROSPERO registration number: CRD42022348304).

## 3. Results

### 3.1. Study Selection

**Figure 1:**
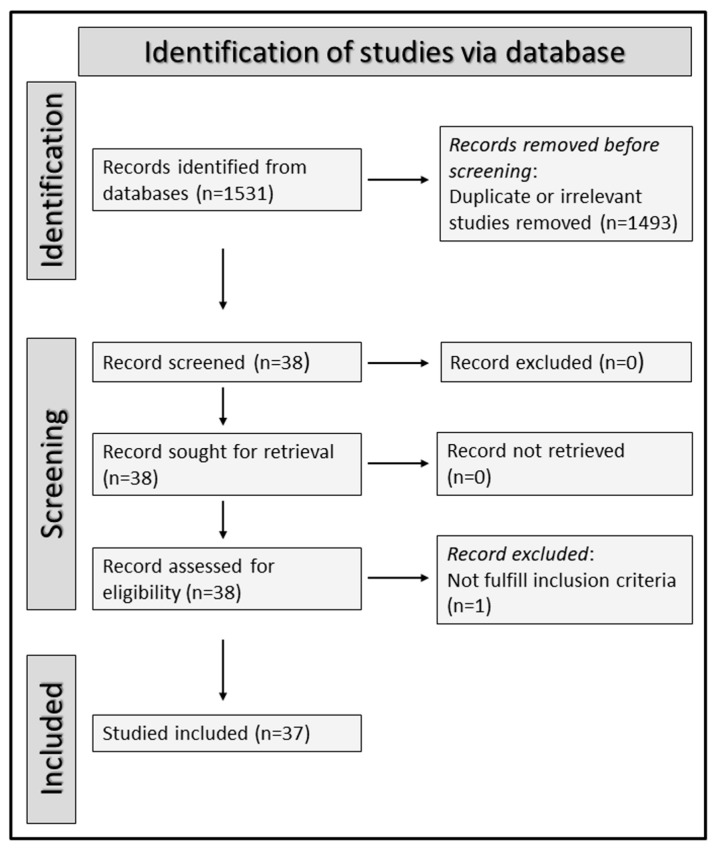
PRISMA 2020 flow diagram.

A total of 1531 articles were initially identified. Of them, 1493 were excluded because they were either duplicates or irrelevant. Following the full-text review of the remaining 38 articles, one was further excluded because it did not fulfil the inclusion criteria (comment letter without providing new data), thus leaving 37 studies for final analysis (Figure 1). Sixteen studies investigated patients with stable COPD [26,27,28,29,30,31,32,33,34,35,36,37,38,39,40,41], 20 investigated patients with AECOPD [42,43,44,45,46,47,48,49,50,51,52,53,54,55,56,57,58,59,60,61], and one investigated both groups [62]. Fourteen studies were conducted in China [32,33,35,36,38,40,41,48,51,53,56,57,58,61]; eleven in Turkey [26,27,28,29,30,31,34,44,49,60,62]; two in Iran [42,47]; two in Israel [43,46]; and one in Indonesia [37], Croatia [39], Greece [50], India [45], Spain [52], Italy [54], Japan [55], and Belgium [59], respectively. Mean or median haemoglobin concentrations were reported as normal in 20 studies [26,27,28,30,31,32,34,35,36,40,41,42,44,47,49,54,59,60,61,62], and low in eight [38,46,48,50,55,56,57,58]. No information regarding haemoglobin concentrations was provided in the remaining nine studies [29,33,37,39,43,45,51,52,53]. Information regarding the mean corpuscular volume was provided in seven studies [26,27,35,42,48,55,59], whereas data on vitamin B_12_ and folic acid status were only reported in three [26,30,54] (Table 1 and Table 2).

### 3.2. RDW and Stable COPD

#### 3.2.1. Studies Selected

Since 2012, 17 studies (four prospective, twelve retrospective, and one with unclear information regarding design) have investigated the clinical role of the RDW in stable COPD [26,27,28,29,30,31,32,33,34,35,36,37,38,39,40,41,62]. Nine studies primarily investigated the relationship between the RDW and the presence and severity of COPD [26,28,29,30,31,34,36,37,39,62], four investigated the association with mortality [27,29,38,40], and the remaining five investigated associations with other parameters, i.e., pulmonary hypertension [30,32,41], pulmonary heart disease [33], and pulmonary embolism [35]. COPD was diagnosed according to the Global Initiative for Chronic Obstructive Lung Disease (GOLD) guidelines in fourteen studies [26,27,28,29,30,31,32,33,34,35,36,39,41,62], and interview questionnaires in one [40], whereas the remaining two did not provide relevant information [37,38] (Table 1).

#### 3.2.2. Risk of Bias

The risk of bias was considered low in 11 studies [26,27,29,30,31,32,33,35,38,40,41], and high in the remaining 6 [28,34,36,37,39,62] (Table 3).

#### 3.2.3. Results of Individual Studies and Syntheses

##### Presence of COPD

Four studies reported the presence of significant differences in RDW values between patients with and without COPD [26,28,30,62]. Sincer et al. investigated 39 patients with stable COPD of at least ten years’ duration from three different centres and 39 age- and sex-matched healthy individuals without overt cardiovascular disease or end-organ injury. RDW values were significantly higher in patients with COPD (16.1 ± 2.5% vs. 13.6 ± 1.3%, *p* < 0.001). In multivariate logistic regression, the RDW was also independently associated with the presence of right ventricular dysfunction on echocardiogram in patients with COPD (OR = 2.20, 95% CI 1.14 to 3.86, *p* = 0.017) after adjusting for re-hospitalisation, forced expiratory volume in the first second (FEV_1_), COPD stage, and FEV_1_/forced vital capacity (FVC). A cut-off RDW value of 17.7% predicted the presence of right ventricular dysfunction with a sensitivity of 70% and a specificity of 93.1% [26]. Gunay et al. retrospectively assessed 178 patients with stable COPD and 50 age- and sex-matched healthy controls. RDW values were significantly higher in patients with COPD (14.6 ± 2.2% vs. 13.7 ± 2.2%, *p* < 0.05) [62]. Yasar et al. investigated 140 patients with COPD, of whom 63 also had metabolic syndrome, and 50 healthy subjects. The RDW values of COPD patients were significantly higher than controls (14.3 ± 1.4% vs. 11.9 ± 1.0%, *p* = 0.005). However, no significant differences in RDW values were observed between COPD patients with and without metabolic syndrome [28]. Ozgul et al. prospectively investigated 175 patients with stable COPD and 210 healthy controls. Seventy COPD patients (40%) and 23 controls (11%) had RDW values > 15.5%, and COPD patients overall had significantly higher RDW values than controls (15.0 ± 2.3% vs. 13.8 ± 2.5%, *p* < 0.001). In multivariate analysis, the RDW was independently associated with the presence of cardiovascular disease (*p* = 0.01) and right ventricular dysfunction (*p* = 0.02) in COPD patients. A cut-off RDW value of 16.9% yielded a sensitivity of 78% and a specificity of 88.7% for right ventricular dysfunction [30]. By contrast, three studies failed to report the presence of significant associations between RDW values and the presence of stable COPD [34,36,39].

##### COPD Severity

Two studies reported significant associations between the RDW and COPD severity [29,31]. Kalemci et al. investigated 153 patients with stable COPD, 39 with mild, 46 with mild-to-moderate, 38 with moderate, and 30 with severe disease. The mean RDW values progressively increased with severity (mild, 13.6 ± 0.4%; mild-to-moderate, 14.1 ± 0.7%; moderate, 15.4 ± 1.1%; severe, 16.8 ± 1.5%; *p* < 0.001 for trend). In multiple logistic regression, after adjusting for age, sex, and hypertension, the RDW was independently associated with severe COPD (odds ratio, OR = 3.67; 95% CI 1.23 to 11.75; *p* < 0.001). A cut-off RDW value of 14.45% yielded a sensitivity of 90% and a specificity of 87% for severe COPD, with an area under the curve (AUC) of 0.948 (95% CI 0.916 to 0.981, *p* < 0.001) [31]. Significant associations between the RDW and GOLD stage were also reported by Tertemiz et al. (r = 0.403, *p* < 0.001) [29]. By contrast, no significant associations between the RDW and COPD severity were reported in three studies [34,37,62].

##### Mortality

Four studies reported significant and independent associations between the RDW and mortality [27,29,38,40]. Seyhan et al. retrospectively assessed 270 patients with stable COPD. In multivariate analysis, the RDW was independently associated with five-year mortality (hazard ratio, HR = 1.12; 95% CI 1.01 to 1.24; *p* = 0.01) after adjusting for age, cardiovascular disease, FEV_1_, PaCO_2_, anaemia, C-reactive protein, pulmonary hypertension, and right ventricular dysfunction [27]. Tertemiz et al. retrospectively studied 385 COPD patients. In multivariate analysis, the RDW was independently associated with nine-year mortality (HR = 1.222, 95% CI 1.153 to 1.295, *p* < 0.01) after adjusting for FEV_1_, six-minute walk test, age, and comorbidities [29]. Lan et al. retrospectively analysed a cohort of 3244 COPD patients admitted to the intensive care unit with critical illness due to non-COPD pathology. In multivariate logistic regression, after adjusting for age, sex, ethnicity, weight, blood pressure, heart rate, respiratory rate, oxygen saturation, haematocrit, platelet count, anion gap, creatinine, bicarbonate, chloride, glucose, urea, white blood cell count, potassium, Simplified Acute Physiology Score II, Sequential Organ Failure Assessment Score, arrhythmias, sepsis, liver disease, vasopressor use, ventilation, renal replacement therapy, acute kidney injury, and anaemia, a significant association was observed between the upper RDW tertile and 28-day mortality (OR = 1.70, 95% CI 1.29 to 2.22, *p* = 0.0001) [38]. Finally, Qiu et al. retrospectively investigated 540 stable COPD patients, 149 with concomitant cardiovascular disease. Multivariate Cox regression analysis showed an independent association between RDW and ten-year mortality (HR = 1.12, 95% CI 1.00 to 1.25, *p* = 0.046) after adjusting for age, sex, smoking, leukocyte count, monocyte count, lymphocyte count, neutrophil count, eosinophil count, basophil count, erythrocytes, haemoglobin, albumin, creatinine, osmolality, cardiovascular disease, blood pressure, body mass index, haematocrit, mean platelet volume, C-reactive protein, alanine aminotransferase, aspartate aminotransferase, alkaline phosphatase, urea, lactate dehydrogenase, uric acid, gamma-glutamyl transferase, glycosylated haemoglobin, high density lipoprotein, neutrophil-to-lymphocyte ratio, lymphocyte-to-monocyte ratio, and systemic inflammation response index [40].

##### Other Clinical Endpoints

Three studies reported significant associations between the RDW and pulmonary hypertension [30,32,41]. In 175 patients with stable COPD, Ozgul et al. reported a significant and positive association between the RDW and the presence of pulmonary hypertension on echocardiogram (r = 0.1, *p* = 0.02) [30]. Yang et al. investigated 174 hospitalised patients with stable COPD without pulmonary hypertension, and 39 COPD patients with pulmonary hypertension. In multivariate analysis, the RDW was independently associated with the presence of pulmonary hypertension (OR = 1.521, 95% CI 1.001 to 2.313, *p* < 0.05) after adjusting for brain natriuretic peptide (BNP), white blood cell count, sex, haemoglobin, and pulmonary artery-to-ascending aorta ratio. The AUC value for RDW was 0.749 ± 0.054. A RDW cut-off value of 14.65 yielded a sensitivity of 69.2% and a specificity of 82.8% for pulmonary hypertension [32]. Similarly, Wang et al. reported that, in multivariable logistic regression, the RDW was independently associated with pulmonary hypertension (OR = 1.054, 95% CI 1.008 to 1.103, *p* = 0.02) after adjusting for GOLD stage, emphysema, PaCO_2_, NT-pro-BNP, and neutrophil-to-lymphocyte ratio. Based on the variables entered in the logistic regression, the authors also constructed a nomogram to predict the probability of pulmonary hypertension. In the predictive model, the pooled AUC was 0.770 (95% CI 0.719 to 0.820) in the training set, and 0.741 (95% CI 0.659 to 0.823) in the validation set [41]. Bai et al. retrospectively investigated 229 COPD patients, including 69 with pulmonary heart disease. In multivariate regression, RDW values were independently associated with pulmonary heart disease (OR = 1.371, 95% CI 1.197 to 1.571, *p* < 0.001) after correcting for white blood cell count, platelet count, mean platelet volume, platelet distribution width, and C-reactive protein. A cut-off RDW-SD value of 48 fL yielded a sensitivity and specificity of 66.7% and 99.4%, respectively, for pulmonary heart disease (AUC = 0.845, *p* < 0.001). Combining the RDW-SD with mean platelet volume increased the AUC value to 0.900 (95% CI 0.846 to 0.954, *p* < 0.001), with a sensitivity and specificity of 76.8% and 99.4%, respectively [33]. Wang J et al. retrospectively studied 125 patients to investigate the relationship between RDW-SD and pulmonary embolism. In multivariate analysis, the RDW-SD was independently associated with pulmonary embolism (OR = 1.188, 95% CI 1.048 to 1.348, *p* = 0.007) after adjusting for albumin, alanine aminotransferase, aspartate aminotransferase, lactate dehydrogenase, and D-dimer. A cut-off RDW-SD value of 44.5% fL yielded a sensitivity and specificity of 80.0% and 64.7%, respectively, for pulmonary embolism (AUC = 0.737, 95% CI 0.635 to 0.839). Furthermore, the combination of RDW and D-dimer further increased the predictive ability, with an AUC value of 0.897, a sensitivity of 87.5%, and a specificity of 83.5% [35].

### 3.3. RDW and AECOPD

#### 3.3.1. Studies Selected

Since 2014, 21 studies (9 prospective and 12 retrospective) have investigated the clinical role of the RDW in AECOPD [42,43,44,45,46,47,48,49,50,51,52,53,54,55,56,57,58,59,60,61,62]. Nine studies primarily investigated the relationship between the RDW and the presence and severity of AECOPD [43,44,45,49,54,56,59,60,62]; seven investigated associations with mortality [42,46,47,48,53,55,60]; and nine investigated associations with other parameters, i.e., hospital readmission and length of stay [46,52,57,58,60], non-invasive mechanical ventilation and oxygen therapy [50], depression and anxiety [51], pulmonary hypertension [56], and pulmonary embolism [61]. AECOPD was diagnosed according to the Global Initiative for Chronic Obstructive Lung Disease (GOLD) guidelines in 13 studies [45,47,48,49,51,54,55,56,57,58,59,60,62], and the Chinese Guidelines for the management of COPD in one [53]. The remaining seven did not provide relevant information regarding the criteria used to diagnose AECOPD [42,43,44,46,50,52,61] (Table 2).

#### 3.3.2. Risk of Bias

The risk of bias was considered low in 11 studies [42,46,47,48,53,55,56,57,58,60,61], and high in the remaining 10 [43,44,45,49,50,51,52,54,59,62] (Table 3).

#### 3.3.3. Results of Individual Studies and Syntheses

##### Presence of AECOPD

Eight studies primarily investigated the relationship between the RDW and the presence and the severity of AECOPD [43,44,45,49,54,56,59,62]. Gunay et al. retrospectively assessed 178 patients with stable COPD, 91 with AECOPD, and 50 age- and sex-matched healthy controls. The RDW values in AECOPD patients were significantly higher than those in patients with stable COPD and controls (median 15.2%; interquartile range, IQR, 2.60% vs. 14.6%, IQR 2.20% vs. 13.7%, IQR 2.25%, respectively; *p* < 0.001 for trend) [62]. Farah et al. reported that RDW values where significantly higher in AECOPD patients compared to stable COPD patients and healthy controls (15.2 ± 1.6% vs. 14.5 ± 1.7% vs. 13.2 ± 0.7%, respectively, *p* < 0.001 for trend) [43]. Koçak et al. also reported significant differences in RDW values between 42 AECOPD patients and 39 stable COPD patients (15.1 ± 0.8% vs. 14.4 ± 0.8%, *p* < 0.001). Similarly, Ragulan et al. reported a significant difference in RDW values between AECOPD and stable COPD patients (*p* < 0.001) [45]. In the study of Marvisi et al., 169 AECOPD patients, 80 stable COPD patients, and 70 healthy subjects were retrospectively assessed. RDW was significantly higher in AECOPD patients than patients with stable COPD (median and IQR, 14.4% (10.3–18.5) vs. 12.0% (10.6–13.2), *p* < 0.001) and healthy subjects (*p* = 0.014). The AUC of the RDW for AECOPD was 0.89 (95% CI 0.84 to 0.93, *p* < 0.0001), with a sensitivity of 83% and a specificity of 82% [54]. Tian et al. reported significant differences in RDW values between 688 patients with AECOPD and 178 patients with stable COPD (median and IQR, 13.5% (12.9–14.6) vs. 13.2% (12.7–13.8), *p* < 0.001) [56]. By contrast, no significant differences in RDW values between patients with stable COPD and AECOPD were reported by Zouaoui Boudjeltia et al. [59].

##### AECOPD Severity

Two studies investigated the association between the RDW and AECOPD severity [60,62]. Koç et al., in 160 hospitalised patients with AECOPD, reported that the RDW was significantly higher in those requiring admission to the intensive care unit compared to those managed in general wards (16.9 ± 3.3% vs. 15.4 ± 2.3%, *p* = 0.003) [60]. By contrast, Gunay et al. did not observe any significant differences in RDW values across stage I to IV patients with AECOPD (*p* = 0.913) [62].

##### Mortality

Seven studies investigated the relationship between RDW and mortality in AECOPD [42,46,47,48,53,55,60]. Rahimirad et al. investigated 225 AECOPD survivors and 75 non-survivors during hospitalisation. In multiple logistic regression, the upper RDW-SD quartile was independently associated with in-hospital mortality (OR = 1.025, *p* = 0.002) after adjusting for age, sex, haemoglobin, and white blood cell count. An RDW-SD cut-off value of 46 fL (upper limit of normal RDW-SD) yielded an AUC value of 0.663 (95% CI 0.597 to 0.729), with a sensitivity of 64% and a specificity of 58% for in-hospital mortality [42]. Epstein et al. conducted a population-based retrospective cohort study on 539 AECOPD patients. The RDW was higher in patients with a composite endpoint of readmission or death at 60 days (15.4 ± 1.9% vs. 14.8 ± 1.8%, *p* = 0.0002). The association between the RDW and the composite endpoint remained significant in multivariate analysis (OR = 1.83, 95% CI 1.22 to 2.74, *p* = 0.0035) after adjusting for acidosis, heart failure, and Charlson comorbidity index. The AUC value, using a cut-off of 14.3%, was 0.61 (95% CI 0.57 to 0.65), with a sensitivity of 68.9% and a specificity of 48.9%. The negative predictive value of normal RDW values was 80.1% (95% CI 74.4 to 85.1) [46]. A total of 1078 AECOPD patients were studied by Torabi et al. In multivariate regression, the RDW was independently associated with in-hospital mortality (OR = 1.02, 95% CI 1.01 to 1.03, *p* < 0.0001) after correcting for white blood cell count, polymorphonuclear leukocytes, mean platelet volume, FEV_1_, and pulmonary artery pressure. The AUC was 0.62, with a sensitivity of 68.9% and a specificity of 48.94% [47]. In a prospective study, Hu et al. investigated the association between RDW and one-year mortality in 442 patients with AECOPD (411 survivors and 31 non-survivors). The RDW was independently associated with mortality (HR = 1.64, 95% CI 1.08 to 2.50, *p* = 0.022) after adjusting for age, body mass index, %FEV_1_, coronary heart disease, heart failure, renal dysfunction, blood pH, pO_2_, and pCO_2_ [48]. In the study by He et al. in 132 AECOPD patients, the RDW was independently associated with one-year mortality in multivariate analysis (OR = 1.796, 95% CI 1.212 to 2.664, *p* = 0.004) after adjusting for age and cancer antigen 125 (CA-125). The AUC of combining the RDW and CA-125 for mortality (0.772, 95% CI 0.690 to 0.855, sensitivity 80%, specificity 67%) was superior to that of the RDW alone (0.691, 95% CI 0.595 to 0.788, sensitivity 77.5%, and specificity 53.3%, using a cut-off value of 12.75%) [53]. By contrast, no significant associations between RDW and 30-day mortality were reported by Sato et al. [55]. Similarly, Koc et al. failed to observe independent associations between RDW and six-month mortality [60].

##### Other Clinical Endpoints

Five studies investigated the association between the RDW and the risk of hospital readmission and/or length of stay [46,52,57,58,60]. In 160 patients hospitalised for AECOPD, Koc et al. did not observe any significant associations between the RDW and the risk of readmission at six months [60]. Negative results were also reported by Garcia-Pachon et al. for readmission at three months [52]. By contrast, Epstein et al. reported independent associations between the RDW and 60-day readmission due to AECOPD (OR = 2.11, 95% CI 1.17 to 3.83, *p* = 0.01) or non-AECOPD causes (OR = 1.72, 95% CI 1.14 to 2.6, *p* = 0.01) after adjusting for acidosis, heart failure, and Charlson comorbidity index [46]. Zhu et al. investigated 239 patients with AECOPD and divided them into three categories according to RDW values during the first and the fourth day after admission: normal RDW, persistently high RDW, and decreasing RDW. In multivariate analysis, a persistently high RDW was independently associated with the risk of 30-day readmission (OR = 3.45, 95% 1.39 to 8.58, *p* = 0.008) after adjusting for the Charlson comorbidity index, coronary heart disease, and pH. By contrast, there were no significant differences in the length of stay across the three groups [57]. The same group conducted another study in 286 hospitalised AECOPD patients divided into three groups according to RDW tertile (<12.8%, 12.9% to 13.6%, and >13.6%). In multivariate logistic regression analysis, the RDW was independently associated with prolonged hospitalisation (OR = 1.400, 95% CI 1.150 to 1.703, *p* = 0.001) after correction for age, white blood cell count, neutrophil count, eosinophil count, FEV_1_%, acidosis, hypercapnia, diabetes, and heart failure. A cut-off value of 13.35% yielded an AUC value of 0.818 (95% CI 0.769 to 0.868), with 83.8% sensitivity and 71.6% specificity for prolonged hospitalisation [58]. Karampitsakos et al., in 160 hospitalised patients with AECOPD, reported that the RDW was independently associated with the need for non-invasive mechanical ventilation (beta coefficient, 0.054; standard error, SE, 0.025; *p* = 0.03) and a history of long-term oxygen therapy (beta coefficient, 0.098; standard error, SE, 0.038; *p* = 0.01) after adjusting for FEV_1_, FVC, haemoglobin, white blood cell count, and eosinophil count [50]. Long et al. prospectively investigated 307 AECOPD patients (195 with and 112 without symptoms of depression and/or anxiety) and 186 healthy controls. RDW values were significantly higher in AECOPD patients with depression and/or anxiety compared to AECOPD without depression and/or anxiety and healthy controls (14.2 ± 1.8% vs. 13.7 ± 1.4% vs. 13.0 ± 1.1%, respectively, *p* < 0.001 for trend). The AUC for depression and/or anxiety in the AECOPD group was 0.570 (95% CI 0.513 to 0.626) using an RDW cut-off value of 14% (sensitivity, 42.0%; specificity, 72.3%) [51]. Tian et al. retrospectively studied 688 patients with AECOPD without pulmonary hypertension and 206 with pulmonary hypertension. In logistic regression, no independent associations were observed between the RDW and pulmonary hypertension (*p* = 0.77) [56]. Finally, Peng et al. retrospectively assessed 262 AECOPD patients with (*n* = 80) and without (*n* = 182) pulmonary embolism. In multivariate regression analysis, the RDW was not independently associated with pulmonary embolism (*p* = 0.152) [61].

## 4. Discussion

Our systematic review identified 37 studies investigating the potential clinical role of the RDW in patients with stable COPD and AECOPD. Overall, the available evidence suggests that this routine haematological parameter used for the diagnosis of anisocytosis may not be particularly useful to discriminate between patients with stable COPD and subjects without COPD. However, it may be a useful biomarker to predict adverse outcomes in patients with stable COPD, with four studies reporting independent associations with mortality [27,29,38,40]. Furthermore, the RDW showed the potential to discriminate between patients with and without AECOPD, with seven out of eight studies reporting significant between-group differences in RDW values [43,44,45,49,54,56,59,62], and to predict adverse outcomes in AECOPD, with five out of seven studies reporting significant independent associations with mortality [42,46,47,48,53,55,60].

The normal volume of a red blood cell typically ranges between 80 fL and 100 fL. However, this value can increase to approximately 150 fL (macrocytosis) or decrease to less than 60 fL (microcytosis) [12,13]. The coexistence of circulating red blood cells with a wide range of volumes is assessed by measuring the RDW, and conventionally defined as anisocytosis. The presence and/or the prognosis of several disease states have been shown to be associated with various degrees of anisocytosis and relatively high RDW values, particularly anaemias secondary to nutritional deficiencies (iron, folic acid, and vitamin B_12_ deficiency), atherosclerotic cardiovascular disease, heart failure, atrial fibrillation, venous thromboembolism, cancer, diabetes, renal failure, liver disease, and lung disorders [12,15,16,17,18,19,20,21,22,23,63,64,65,66,67,68]. Notably, the absolute RDW values and differences between patients with stable COPD and AECOPD and control groups in the studies identified in our systematic review are similar to those reported in other disease states, e.g., cardiovascular disease. For example, in a study by Lippi et al., the RDW values in patients with and without acute coronary syndrome were 15.1% vs. 13.5% (*p* < 0.001) [69] Similarly, Ma et al. reported that patients with angiographically documented coronary artery disease had significantly higher RDW values than subjects without (13.0% vs. 12.7%, *p* = 0.001) [70].

The results of our systematic review expand the potential clinical applications of the RDW to COPD; however, the exact mechanisms responsible for the development of anisocytosis in this group, particularly patients at risk of adverse outcomes, remain elusive. By and large, as previously discussed, anisocytosis is secondary to a reduced clearance of older red blood cells by macrophages located in the spleen and the liver and/or by a reduced production of red blood cell in the bone marrow [12,13]. Though the role of an impaired function of splenic macrophages has been poorly investigated in the context of COPD, there is increasing evidence that the liver is the primary organ involved in red blood cell clearance [71]. Notably, liver disease is particularly common in patients with COPD, and the consequent impairment in red blood cell clearance might theoretically account for the higher RDW in this group [72,73,74]. However, in our systematic review, liver disease represented a significant comorbidity only in one study [38]. An impaired production of red blood cells, with a consequent increase in RDW values, would be expected in the context of anaemia secondary to iron, vitamin B_12_, or folic acid deficiency [12,13]. However, reduced haemoglobin was documented in only eight studies identified in our review [38,46,48,50,55,56,57,58], and no information regarding haemoglobin status was provided in the other eight [29,33,37,39,43,45,51,52,53]. Furthermore, data on the mean corpuscular volume were only provided in seven studies [26,27,35,42,48,55,59], whereas information regarding vitamin B_12_ and folic acid status was limited to three studies [26,30,54]. Notably, other conditions that are commonly associated with an increased RDW, i.e., cardiovascular disease and diabetes, were reported in 19 out of 37 studies [26,27,29,31,32,33,35,36,38,40,42,46,48,56,57,58,59,60,61].

A common denominator in patients with COPD, atherosclerotic cardiovascular disease, and diabetes is the presence of local and systemic oxidative stress and increased inflammation [75,76,77,78,79,80,81,82]. Notably, there is evidence that inflammatory cytokines can inhibit the maturation of erythroid cells in the bone marrow, with the consequent release into the circulation of immature erythrocytes. This leads to an increased heterogeneity in the volume of circulating red blood cells, and higher RDW values [83,84]. The potential link between inflammation and anisocytosis is supported by the significant and positive associations reported in four studies in our systematic review between the RDW and the inflammatory markers, C-reactive protein, and neutrophil-to-lymphocyte ratio (Table 1 and Table 2) [27,30,54,62]. Furthermore, a state of increased oxidative stress in red blood cells can alter their mechanical properties and structure, consequently favouring an increase in the RDW [85,86]. Though these data suggest that an increase in the RDW in COPD might reflect a combined state of respiratory and extra-pulmonary organ dysfunction, with an associated state of increased inflammation and oxidative stress, more research is warranted to investigate the mechanisms involved in the reported increase in the RDW in patients with COPD. At the same time, larger, appropriately designed prospective studies are also needed to confirm the overall results of our systematic review, particularly in terms of predicting adverse clinical outcomes. Ideally, such studies should investigate COPD and AECOPD patients with different clinical severity, comorbid status, and ethnicity, and investigate the predictive capacity of the RDW, singly or in combination with other biomarkers or scoring tools, over relatively long follow-up periods. In this context, three studies in our meta-analysis have reported that the combination with other biomarkers significantly increased the AUC value of the RDW [33,35,53].

The strengths of our systematic review include the comprehensive assessment of the diagnostic and the prognostic capacity of the RDW, as well as the strength of the association with other relevant clinical parameters, in patients with both stable COPD and AECOPD. Furthermore, the risk of bias was considered low in the majority of studies, 22 out of 27. Limitations include the lack of assessment by meta-analysis, given the heterogeneity of the studied populations, endpoints, and their characterisation; issues with the generalisability of the results secondary to the paucity of data from African, European, and American populations; and, as previously discussed, the lack of information provided in most studies regarding the presence of anaemia, vitamin B_12_, and folic acid status, important determinants of anisocytosis *per se*.

## 5. Conclusions

Our systematic review has shown the potential clinical role of the RDW, a routine haematological parameter that indicates the presence of anisocytosis, in discriminating between COPD patients with and without AECOPD, and, particularly, in predicting adverse outcomes in patients with COPD and AECOPD. Appropriately designed prospective studies are warranted to confirm these findings and justify the introduction of the RDW for routine clinical use in the management of COPD.

## Figures and Tables

**Table 1:** Characteristics of studies investigating associations between RDW and clinical endpoints in patients with stable COPD.

First Author, Year, Country [Ref]	Study Design	N	COPD Diagnosis	Main Comorbidities MCV Haemoglobin Vitamin B_12_ Folate	Main Results	Significant Associations between RDW and Other Parameters
Sincer I, 2012, Turkey [26]	P	39	GOLD	Hypertension Diabetes Normal MCV Normal haemoglobin B_12_ deficiency EC Folate deficiency EC	RDW significantly higher in COPD patients than healthy controls RDW independently associated with right ventricular dysfunction in COPD patients (RDW cut-off = 17.7%)	TAPSE (r = −0.538, *p* < 0.001) Systolic velocity (r = −0.532, *p* = 0.004)
Seyhan EC, 2013, Turkey [27]	R	270	GOLD	Hypertension Cardiovascular disease Normal MCV Normal haemoglobin B_12_ status NR Folate status NR	RDW independently associated with five-year mortality in COPD patients	COPD duration (r = 0.21, *p* = 0.02) Long-term O_2_ therapy (r = 0.16, *p* = 0.03) PaCO_2_ (r = 0.17, *p* = 0.04) Albumin (r = −0.22, *p* = 0.02) Haemoglobin (r = −0.18, *p* = 0.04) MCV (r = −0.37, *p* < 0.001) CRP (r = 0.21, *p* = 0.008) RVD (r = 0.25, *p* < 0.001) PAH (r = 0.14, *p* = 0.03) LVEF (r = −0.15, *p* = 0.02) RVEDD (r = 0.17, *p* = 0.03) LVEDD (r = 0.15, *p* = 0.04)
Gunay E, 2014, Turkey, [62]	R	178	GOLD	NR MCV NR Normal haemoglobin B_12_ status NR Folate status NR	RDW significantly higher in COPD patients than healthy controls RDW not significantly associated with GOLD stage	NLR (r = 0.268, *p* < 0.001)
Yasar Z, 2015, Turkey [28]	R	140	GOLD	NR MCV NR Normal haemoglobin B_12_ status NR Folate status NR	RDW significantly higher in COPD patients than healthy controls RDW not significantly associated with the presence of metabolic syndrome in COPD patients	NR
Tertemiz KC, 2016, Turkey [29]	R	385	GOLD	Cardiovascular disease MCV NR Haemoglobin NR B_12_ status NR Folate status NR	RDW independently associated with nine-year mortality in COPD patients RDW significantly associated with GOLD stage	6MWT (r−0.279, *p* < 0.001) %FEV_1_ (r = −0.290, *p* < 0.001) %FVC (r = −0.285, *p* < 0.001) FEF25–75% (r = −0.303, *p* < 0.01) %PEF (r = −0.227, *p* < 0.01) O_2_ saturation (r = −0.260, *p* < 0.001) Age (r = 0.182, *p* < 0.001) BODE index (r = 0.407, *p* < 0.001)
Ozgul G, 2017, Turkey [30]	P	175	GOLD	NR MCV NR Normal haemoglobin B_12_ deficiency EC Folate deficiency EC	RDW significantly higher in COPD patients than healthy controls RDW independently associated with cardiovascular disease and right ventricular dysfunction (RDW cut-off = 16.9%)	CRP (r = 0.27, *p* = 0.001) Haemoglobin (r = −0.26, *p* < 0.001) Albumin (r = −0.23, *p* = 0.04) PAH (r = 0.1, *p* = 0.02)
Kalemci S, 2018, Turkey [31]	R	153	GOLD	Hypertension Diabetes MCV NR Normal haemoglobin B_12_ status NR Folate status NR	RDW independently associated with severe disease in COPD patients (RDW cut-off = 14.45%)	NR
Yang J, 2019, China [32]	R	213	GOLD	Hypertension Diabetes IHD MCV NR Normal haemoglobin B_12_ status NR Folate status NR	RDW independently associated with PAH in COPD patients (RDW cut-off = 14.65%)	BNP (r = 0.513, *p* < 0.001) PASP (r = 0.390, *p* = 0.014) PA:A (r = 0.502, *p* < 0.001)
Bai Y, 2020, China [33]	R	229	GOLD	Hypertension Diabetes MCV NR Haemoglobin NR B_12_ status NR Folate status NR	RDW independently associated with pulmonary heart disease in COPD patients (RDW-SD cut-off = 48 fL)	*COPD * %FEV_1_ (r = −0.838, *p* < 0.001) PAP (r = 0.734, *p* < 0.001) RVS (r = 0.546, *p* < 0.001) *COPD with pulmonary heart disease * %FEV_1_ (r = −0.768, *p* < 0.001) PAP (r = 0.820, *p* < 0.001) RVS (r = 0.845, *p* < 0.001)
Çilingir BM, 2020, Turkey [34]	R	201	GOLD	NR MCV NR Normal haemoglobin B_12_ status NR Folate status NR	RDW not significantly different between COPD patients and healthy controls RDW not significantly associated with disease severity in patients with COPD	NR
Wang J, 2020, China [35]	R	125	GOLD	Hypertension Diabetes Normal MCV Normal haemoglobin B_12_ status NR Folate status NR	RDW independently associated with pulmonary embolism in patients with COPD (RDW-SD cut-off = 44.5 fL)	NR
Huang Y, 2021, China [36]	P	100	GOLD	Hypertension Diabetes MCV NR Normal haemoglobin B_12_ status NR Folate status NR	RDW not significantly different between COPD patients and healthy controls	NR
Martunis M, 2021, Indonesia [37]	NR	30	NR	NR MCV NR Haemoglobin NR B_12_ status NR Folate status NR	RDW not significantly associated with disease severity in patients with COPD	NR
Lan W, 2022, China [38]	R	3244	NR	Heart failure Arrhythmia Hypertension Diabetes Renal failure Liver disease Sepsis Anaemia MCV NR Low haemoglobin B_12_ status NR Folate status NR	RDW independently associated with 28-day mortality	Significant differences across RDW tertiles for: Age (*p* = 0.02) Ethnicity (*p* = 0.03) Blood pressure (*p* < 0.001) Respiratory rate (*p* = 0.001) Temperature (*p* = 0.04) SpO_2_ (*p* = 0.03) Haemoglobin (*p* < 0.001) White blood cells (*p* = 0.02) Anion gap (*p* < 0.001) Bicarbonate (*p* = 0.002) Creatinine (*p* < 0.001) SOFA (*p* < 0.001) SAPS II (*p* < 0.001) Heart failure (*p* < 0.001) Arrhythmias (*p* < 0.001) Diabetes (*p* < 0001) Renal failure (*p* < 0.001) Renal replacement therapy (*p* < 0.001) Liver disease (*p* < 0.001) Sepsis (*p* < 0.001) Anaemia (*p* < 0.001) Ventilation (*p* < 0.001)
Ljubičić D, 2022, Croatia [39]	P	61	GOLD	NR MCV NR Haemoglobin NR B_12_ status NR Folate status NR	RDW not significantly different between COPD patients and healthy controls	None
Qiu Y, 2022, China [40]	R	540	Interview questionnaires	Cardiovascular disease MCV NR Normal haemoglobin B_12_ status NR Folate status NR	RDW independently associated with the presence of cardiovascular disease in patients with COPD RDW independently associated with the risk of ten-year mortality in patients with COPD	NR
Wang N, 2022, China [41]	R	527	GOLD	NR MCV NR Normal haemoglobin B_12_ status NR Folate status NR	RDW independently associated with the presence of pulmonary hypertension in patients with COPD	NR

Legend: 6MWT, six-minute walking test; ALT, alanine aminotransferase; AST, aspartate aminotransferase; BMI, body mass index; CRP, C-reactive protein; EC, exclusion criterion; FEV_1_, forced expiratory volume in the 1st second; FEF, forced mid-expiratory flow rate; FEF 25–75%, maximum expiratory flow in the middle half of the forced expiratory manoeuvre; FVC, forced vital capacity; GOLD, Global Initiative for Chronic Obstructive Lung Disease guidelines; IHD, ischaemic heart disease; LDH, lactate dehydrogenase; LVEDD, left ventricular end diastolic diameter; LVEF, left ventricular ejection fraction; MCV, mean corpuscular volume; NLR, neutrophil-to-lymphocyte ratio; NR, not reported; *p*, prospective; PA:A, pulmonary artery-to-ascending aorta ratio; PAH, pulmonary arterial hypertension; PAP, pulmonary artery pressure; PASP, pulmonary artery systolic pressure; PE, pulmonary embolism; PEF, peak expiratory flow; PH, pulmonary hypertension; PHD, pulmonary heart disease; PYI, pack-years index; RDW, red cell distribution width; RDW-SD, standard deviation of the RDW; RVD, right ventricular dysfunction; R, retrospective; RVEDD, right ventricular end diastolic diameter; RVF, right ventricular failure; RVS, right ventricular size; SOFA, sequential organ failure assessment score; SAPS II, simplified acute physiology score; sPAP, systolic pulmonary artery pressure; TAPSE, tricuspid annular plane systolic excursion.

**Table 2:** Characteristics of studies investigating associations between RDW and clinical endpoints in patients with acute exacerbation of COPD (AECOPD).

First Author, Year, Country [Ref]	Study Design	N	COPD Diagnosis	Main Comorbidities MCV Haemoglobin Vitamin B_12_ Folate	Main Result	Significant Association between RDW and Other Parameters
Gunay E, 2014, Turkey [62]	R	269	GOLD	NR MCV NR Normal haemoglobin B_12_ status NR Folate status NR	RDW significantly higher in patients with AECOPD when compared to COPD patients with stable disease and healthy controls No significant differences in RDW between disease severity classes in AECOPD	NLR (r = 0.292, *p* = 0.005)
Rahimirad S, 2016, Iran [42]	R	330	NR	Diabetes Hypertension IHD Heart failure Normal MCV Normal haemoglobin B_12_ status NR Folate status NR	RDW independently associated with in-hospital mortality in patients with AECOPD (RDW-SD cut-off = 46 fL)	Haemoglobin (r = −1.42, *p* = 0.01)
Farah R, 2017, Israel [43]	P	85	NR	NR MCV NR Haemoglobin NR B_12_ status NR Folate status NR	RDW significantly higher in patients with AECOPD when compared to COPD patients with stable disease and healthy controls	None
Koçak MZ, 2017, Turkey [44]	R	81	NR	NR MCV NR Normal haemoglobin B_12_ status NR Folate status NR	RDW significantly higher in patients with AECOPD when compared to COPD patients with stable disease	WBC (r = 0.244, *p* = 0.029)
Ragulan R, 2017, India [45]	P	135	GOLD	NR MCV NR Haemoglobin NR B_12_ status NR Folate status NR	RDW significantly higher in patients with AECOPD when compared to COPD patients with stable disease	Sex (males > females, *p* < 0.001)
Epstein D, 2018, Israel [46]	R	539	NR	Diabetes Hypertension Anaemia Heart failure MCV NR Low haemoglobin B_12_ status NR Folate status NR	RDW independently associated with 60-day readmission due to AECOPD, 60-day readmission from any reason, and 60-day composite endpoint of readmission or death (RDW cut-off = 14.3%)	NR
Torabi M, 2018, Iran [47]	P	1078	GOLD	NR MCV NR Normal haemoglobin B_12_ status NR Folate status NR	RDW independently associated with in-hospital mortality in patients with AECOPD	NR
Hu GP, 2019, China [48]	P	442	GOLD	Hypertension IHD Heart failure Normal MCV Low haemoglobin B_12_ status NR Folate status NR	RDW independently associated with one-year mortality in patients with AECOPD	NR
Şahin F, 2019, Turkey [49]	R	250	GOLD	NR MCV NR Normal haemoglobin B_12_ status NR Folate status NR	RDW significantly higher in patients with AECOPD when compared to COPD patients with stable disease and healthy controls	NR
Karampitsakos T, 2020, Greece [50]	P	160	NR	NR MCV NR Low haemoglobin B_12_ status NR Folate status NR	RDW independently associated with the need for non-invasive mechanical ventilation and long-term oxygen therapy in patients with AECOPD	NR
Long J, 2020, China [51]	P	307	GOLD	NR MCV NR Haemoglobin NR B_12_ status NR Folate status NR	RDW significantly higher in patients with AECOPD and depression/anxiety compared to patients with AECOPD without depression/anxiety and healthy controls (RDW cut-off = 14.0%)	HAMA score (r = 0.116, *p* = 0.042) HAMD score (*p* = 0.156, *p* = 0.006)
Garcia-Pachon E, 2021, Spain [52]	P	106	NR	NR MCV NR Haemoglobin NR B_12_ status NR Folate status NR	RDW not significantly associated with risk of three-month readmission in patients with AECOPD	NR
He F, 2021, China [53]	R	132	Chinese COPD guidelines	NR MCV NR Haemoglobin NR B_12_ status NR Folate status NR	RDW independently associated with one-year mortality in patients with AECOPD (RDW cut-off = 12.75%)	NR
Marvisi M, 2021, Italy [54]	R	249	GOLD	NR MCV NR Normal haemoglobin B_12_ deficiency EC Folate deficiency EC	RDW significantly higher in patients with AECOPD when compared to COPD patients with stable disease and healthy subjects	CRP (r = 0.375, *p* < 0.01) CAT Score (r = 0.811, *p* < 0.01) Number of exacerbations (r = 0.538, *p* = 0.002) GOLD score (r = 0.547, *p* = 0.05) Number of packs smoked (r = 0.372, *p* < 0.01)
Sato K, 2021, Japan [55]	R	195	GOLD	NR Normal MCV Low haemoglobin B_12_ status NR Folate status NR	RDW not independently associated with 30-day mortality in patients with AECOPD	NR
Tian F, 2021, China [56]	R	1072	GOLD	Diabetes IHD Hypertension Stroke MCV NR Low haemoglobin B_12_ status NR Folate status NR	RDW significantly higher in AECOPD patients that patients with stable COPD RDW independently associated with pulmonary hypertension in AECOPD patients	NT-pro BNP (r = 0.359, *p* < 0.001)
Zhu M (a), 2021, China [57]	R	239	GOLD	Hypertension Diabetes IHD Heart failure MCV NR Low haemoglobin B_12_ status NR Folate status NR	Persistently high RDW on admission independently associated with 30-day readmission compared to decreasing RDW and normal RDW in AECOPD patients No significant differences in length of stay between the three groups	NR
Zhu M (b), 2021, China [58]	R	286	GOLD	Hypertension Diabetes Atrial fibrillation Heart failure MCV NR Low haemoglobin B_12_ status NR Folate status NR	RDW independently associated with length of stay in patients with AECOPD (RDW cut-off = 13.35%)	Haemoglobin, (r = −0.470, *p* < 0.001) %FEV_1_ (r = −0.142, *p* = 0.016)
Zouaoui Boudjeltia K, 2021, Belgium [59]	P	73	GOLD	Hypertension Diabetes Normal MCV Normal haemoglobin B_12_ status NR Folate status NR	RDW not significantly different between patients with AECOPD and patients with stable COPD	NR
Koç C, 2022, Turkey [60]	P	160	GOLD	Hypertension Diabetes Heart failure IHD MCV NR Normal haemoglobin B_12_ status NR Folate status NR	RDW significantly higher in AECOPD patients admitted to ICU compared to those not admitted RDW not significantly different in AECOPD readmitted vs. not readmitted at six months RDW not independently associated with six-month mortality	NR
Peng G, 2022, China [61]	R	262	NR	Diabetes IHD MCV NR Normal haemoglobin B_12_ status NR Folate status NR	RDW not independently associated with pulmonary embolism in patients with AECOPD	NR

Legend: CAT, COPD assessment test; CRP, C-reactive protein; EC, exclusion criterion; FEV_1_, forced expiratory volume in the first second; GOLD, Global Initiative for Chronic Obstructive Lung Disease guidelines; HAMA, Hamilton anxiety rating scale; HAMD, Hamilton rating scale for depression; IHD, ischaemic heart disease; MCV, mean corpuscular volume; NLR, neutrophil-to-lymphocyte ratio; NT-pro BNP, N-terminal pro brain natriuretic peptide; NR, not reported; RDW, red cell distribution width; RDW-SD, standard deviation of the RDW; WBC, white blood cell count.

**Table 3:** The Joanna Briggs Institute critical appraisal checklist.

Study	Were the Groups Comparable Cther than the RDW?	Were the Same Criteria Used for Identification of Cases and Controls?	Was Exposure Measured in a Valid and Reliable Way? Was is Capitalized Because It Is the First Word of the Title. More Information on Capita	Was Exposure Measured in the Same Way for Cases and Controls?	Were Confounding Factors Identified?	Were Strategies to Deal with Confounding Factors Stated?	SWere Outcomes Assessed in a Standard, Valid, and Reliable Way for Cases and Controls?	Was the Exposure Period of Interest Long Enough to Be Meaningful?	Was Appropriate Statistical Analysis Used?	Risk of Bias
Sincer I [26]	No	Yes	Yes	Yes	Yes	Yes	Yes	Yes	Yes	Low
Seyhan EC [27]	No	Yes	Yes	Yes	Yes	Yes	Yes	Yes	Yes	Low
Gunay E [62]	No	Yes	Yes	Yes	No	No	Yes	Yes	No	High
Yasar Z [28]	No	Yes	Yes	Yes	No	No	Yes	Yes	No	High
Tertemiz KC [29]	No	Yes	Yes	Yes	Yes	Yes	Yes	Yes	Yes	Low
Ozgul G [30]	No	Yes	Yes	Yes	Yes	Yes	Yes	Yes	Yes	Low
Kalemci S [31]	No	Yes	Yes	Yes	Yes	Yes	Yes	Yes	Yes	Low
Yang J [32]	No	Yes	Yes	Yes	Yes	Yes	Yes	Yes	Yes	Low
Bai Y [33]	No	Yes	Yes	Yes	Yes	Yes	Yes	Yes	Yes	Low
Çilingir BM [34]	No	Yes	Yes	Yes	No	No	Yes	Yes	No	High
Wang J [35]	No	Yes	Yes	Yes	Yes	Yes	Yes	Yes	Yes	Low
Huang Y [36]	No	Yes	Yes	Yes	No	No	Yes	Yes	No	High
Martunis M [37]	No	Yes	No	Yes	No	No	Yes	Yes	No	High
Lan W [38]	No	Yes	No	Yes	Yes	Yes	Yes	Yes	Yes	Low
Ljubičić D [39]	No	Yes	Yes	Yes	No	No	Yes	Yes	No	High
Qiu Y [40]	No	Yes	No	Yes	Yes	Yes	Yes	Yes	Yes	Low
Wang N [41]	No	Yes	Yes	Yes	Yes	Yes	Yes	Yes	Yes	Low
Rahimirad S [42]	No	Yes	No	Yes	Yes	Yes	Yes	Yes	Yes	Low
Farah R [43]	No	Yes	No	Yes	No	No	Yes	Yes	No	High
Koçak MZ [44]	No	Yes	No	Yes	No	No	Yes	Yes	No	High
Ragulan R [45]	No	Yes	Yes	Yes	No	No	Yes	Yes	No	High
Epstein D [46]	No	Yes	No	Yes	Yes	Yes	Yes	Yes	Yes	Low
Torabi M [47]	No	Yes	Yes	Yes	Yes	Yes	Yes	Yes	Yes	Low
Hu GP [48]	No	Yes	Yes	Yes	Yes	Yes	Yes	Yes	Yes	Low
Şahin F [49]	No	Yes	Yes	Yes	No	No	Yes	Yes	No	High
Karampitsakos T [50]	No	Yes	No	Yes	No	No	Yes	Yes	No	High
Long J [51]	No	Yes	Yes	Yes	No	No	Yes	Yes	No	High
Garcia-Pachon E [52]	No	Yes	No	Yes	No	No	Yes	Yes	No	High
He F [53]	No	Yes	Yes	Yes	Yes	Yes	Yes	Yes	Yes	Low
Marvisi M [54]	No	Yes	Yes	Yes	No	No	Yes	Yes	No	High
Sato K [55]	No	Yes	Yes	Yes	Yes	Yes	Yes	Yes	Yes	Low
Tian F [56]	No	Yes	Yes	Yes	Yes	Yes	Yes	Yes	Yes	Low
Zhu M (a) [57]	No	Yes	Yes	Yes	Yes	Yes	Yes	Yes	Yes	Low
Zhu M (b) [58]	No	Yes	Yes	Yes	Yes	Yes	Yes	Yes	Yes	Low
Zouaoui Boudjeltia K [59]	No	Yes	Yes	Yes	No	No	Yes	Yes	No	High
Koç C [60]	No	Yes	Yes	Yes	Yes	Yes	Yes	Yes	Yes	Low
Peng G [61]	No	Yes	No	Yes	Yes	Yes	Yes	Yes	Yes	Low

## Data Availability

The data that support the findings of this systematic review and meta-analysis are available from the corresponding author, A.Z., upon reasonable request.

## Supplementary Materials

The following are available online at https://www.mdpi.com/article/10.3390/jcm11195642/s1, Table S1: PRISMA 2020 abstract checklist; Table S2: PRISMA 2020 manuscript checklist.
